# Health Services for Gender-Based Violence: Médecins Sans Frontières Experience Caring for Survivors in Urban Papua New Guinea

**DOI:** 10.1371/journal.pone.0156813

**Published:** 2016-06-10

**Authors:** Kamalini Lokuge, Meggy Verputten, Maryanne Ajakali, Bianca Tolboom, Grace Joshy, Katherine A. Thurber, Daisy Plana, Steven Howes, Anastasia Wakon, Emily Banks

**Affiliations:** 1 National Centre for Epidemiology and Population Health, Research School of Population Health, the Australian National University, Acton, Australia; 2 Médecins Sans Frontières (MSF)-UK, London, United Kingdom; 3 Femili PNG, Lae, Papua New Guinea; 4 MSF-Operational Centre Amsterdam, Amsterdam, the Netherlands; 5 Angau Hospital, National Department of Health, Lae, Papua New Guinea; 6 MSF-Operational Centre Papua New Guinea Programme, Port Moresby, Papua New Guinea; 7 Development Policy Centre, Crawford School of Public Policy, the Australian National University, Acton, Australia; Johns Hopkins Bloomberg School of Public Health, UNITED STATES

## Abstract

**Background:**

Levels of gender-based violence in Papua New Guinea (PNG) are high; health services for survivors are limited. Evidence from the few existing health services for survivors can inform improvements in care in this and similar settings.

**Methods:**

Médecins Sans Frontières supported health services for survivors in Lae, PNG from 2008–2013. Routine monitoring data from August 2010-April 2013 were used to describe patient and service characteristics.

**Results:**

5,892 individuals received care over 6,860 presentations, the majority self-referred or referred by friends and family. Presentations were attributed to intimate partner violence(62%), non-partner sexual violence(15%), other forms of violence(3%), and past (but not current) violence(21%). 97% were female; an estimated 4.9% (95%CI:4.8–5.0%) of females resident in the catchment area presented to the programme during the 2.8years analysed. Of presentations for non-partner sexual violence, 79% knew their abuser and 50% were children <16 years. 92% of presentations reporting current violence received medical treatment for injuries. The majority of patients who received multiple counselling sessions reported improved functioning and decreased severity of psycho-social complaints.

**Conclusions:**

Community awareness of the availability of free, best-practice, accessible, confidential medical and counselling services for sexual and gender-based violence in Lae, PNG resulted in many survivors presenting for care. High levels of ongoing intimate partner violence and child sexual abuse by known abusers indicates that alongside comprehensive medical care, access to effective services in non-health sectors such as policing, protection and legal services are needed if survivors are to escape the cycle of violence.

## Introduction

Existing data suggest that levels of gender-based violence in Papua New Guinea (PNG) are extremely high. Past studies have found two thirds of women report intimate partner violence, and over half report being forced into sex against their will, mostly by men known to them [1]. A more recent study in the Bougainville Autonomous region of Papua New Guinea in 2012–2013 had similar findings, with 3 in 4 women surveyed reporting ever having experienced intimate partner violence. In this same survey, 16% reported having ever experienced non-partner rape. Of these, 8% reported such rape within the previous 12 months. This survey data was also part of a 2012 multi-country cross-sectional study of men’s perpetration of gender-based violence. The proportion of ever-partnered men who reported perpetrating physical violence, sexual violence or both against a partner was 80.0% in the sample from Bougainville, PNG–the highest of all 19 countries included in the study [2, 3]. Similarly, the prevalence of perpetration of non-partner single perpetrator rape (26.6%) and multiple perpetrator rape (14.1%) was also highest in the PNG sample.

In conjunction with the burden gender-based violence imposes on the health and wellbeing of women and children [4, 5], health services for survivors are limited. PNG’s health system has been described by the World Health Organization as being in a state of ‘slow breakdown and collapse’ [6], meaning that survivors of gender-based violence are faced with significant challenges in accessing medical care. Survivors also require appropriate counselling [7], but the availability of such services is even lower than that of medical services. There is very limited information on the best approaches to providing care in PNG or similar resource-constrained settings [8, 9], despite the fact that these are often where the greatest levels of gender-based violence occur [10]. There is an urgent need for evidence to inform the pragmatic delivery of effective medical care and counselling for survivors in such settings.

Médecins Sans Frontières (MSF) is an international, independent, non-governmental medical humanitarian organisation that assists populations affected by armed conflict, epidemics, natural disasters, and exclusion from healthcare. An assessment of health services in PNG in 2006 by the Amsterdam Operational Centre of MSF(MSF-OCA) identified effective services for gender-based violence as a major need throughout the country [11], including in Lae, the second largest city in PNG. At the time of the MSF assessment, the Family Support Centre (FSC) at the National Department of Health’s provincial hospital in Lae (Angau Hospital) had a core group of staff committed to addressing gender-based violence. MSF-OCA provided medical, management, human resource and material support to the FSC for the provision of medical care and counselling services from late 2007 until June 2013. This experience provides a unique opportunity to gain insights into the provision and use of health services for survivors of gender-based violence. Understanding who utilises services for survivors, what challenges exist in delivering these services, the factors promote retention in care, and if and how patients benefit from that care is critical to inform the development of effective services in this and similar settings. The specific objectives of this analysis were therefore to:

1. Describe the demographic characteristics of patients presenting to the FSC, Lae;
2. Describe the characteristics of event(s) associated with presentation, including type of violence and reported perpetrator;
3. Describe the medical care and counselling received by these patients, including the outcomes of counselling care, and; Investigate which recorded patient and service factors were associated with service use, including timeliness and repeated presentation.

## Materials and Methods

### Programme objectives

From early 2008, the programme objectives were provision of medical and counselling care to survivors of intimate partner and sexual violence in Lae and surrounding districts. These objectives remained consistent until project handover to local management in June 2013. Services were provided through the FSC throughout this period. In addition, from April to October 2012, medical care was also provided through selected urban health centres within Lae.

MSF-supported services were free of charge and could be accessed directly by patients without requiring referral documentation. Services included the following components of care: medical treatment for injuries; provision of tetanus and hepatitis B vaccination; emergency contraception; HIV testing, post-exposure prophylaxis for HIV; treatment and prevention of other sexually transmitted infections; provision of medical certificates documenting the service provider’s findings of the medical examination; referral for further medical treatment or other services; and therapeutic counselling services based on principles derived from brief trauma-focused therapy as outlined in the MSF-OCA mental health guidelines [12]. Counselling services included psychological first aid (providing reassurance and normalizing emotions and feelings after a traumatic incident), and basic general and informative counselling to support the patient to express concerns and identify solutions. Counsellors also assessed client safety, and if required discussed safety measures. For patients who presented following sexual assault pre and post testing counselling and adherence counselling for HIV prophylaxis were also provided when indicated.

MSF-OCA is a medical organisation, and as such, provision of non-medical services such as legal and protection support was beyond the scope of the programme.

Community awareness regarding health services for survivors was raised through outreach activities including disseminating printed health promotion materials, radio messages and participative dialogue with community groups.

### Data collection and recording

These analyses were based on data routinely collected from all patients (or carers in the case of young children) during provision of services from August 2010 to April 2013. Data were de-identified by the MSF program data manager responsible for entering and analysing data for routine patient care and program purposes and provided in this de-identified form for the analyses undertaken in this paper.

Reported violence experienced during the event associated with presentation was categorised as:

1. Intimate partner violence, including sexual violence, physical violence, or both perpetrated by an intimate partner (IPV) with or without associated ongoing emotional/psychological violence;
2. Non-partner sexual violence (non-partner SV);
3. Violence other than IPV or non-partner SV (other violence); or
4. Patients with no current physical injuries and not reporting a specific event of violence associated with presentation, and therefore requiring counselling only (past violence).

The above definitions and terminology were utilised when presenting results, including in the tables. All the above reported violence relates to current violence associated with the specific presenting event(s), except where ‘past violence’ is specified. Where specific event(s) of violence were reported (categories 1–3 above), the reported perpetrator of that violence was also recorded. Reliable data on severity of injury were not collected; therefore type of medical treatment was used as a proxy measure. Where specific event(s) of sexual violence were reported, information was also collected on the type of sexual violence, categorised as rape or other (including attempted rape, child sexual abuse, sexual exploitation and sexual harassment); number of perpetrators; and frequency of such violence.

Multiple visits to the service related to the same event(s) of violence during the study period were recorded as a single presentation. However, if the same individual presented for a second or additional episode following new event(s) of violence during the study period, each was recorded as a new presentation.

Although patients may have received multiple medical interventions, only the primary intervention as defined by health staff was recorded. Data were also collected on counselling services provided. A counselling session was defined as a face-to face session with the counsellor on a single day of a duration as required by the client. The underlying precipitating event associated with referral for counselling was categorised as domestic violence or discord (intimate partner physical violence or non-violent abuse including arguments and threats, current or past), sexual violence or abuse (current or past events of such violence) or other (including economic stresses and non-intimate partner household conflict). Data included the number of counselling sessions received per presentation, severity of main psychosocial presenting complaint (which included mood disorders, anxiety related complaints, family related problems) and functional status (measured using indicators related to the patient’s capacity to undertake normal daily activities including self-care, caring for children, household activities such as cooking and attendance at work). Both were self-rated on a scale from 1–10, with 1 = most severe) and recorded at each counselling session. These measures were developed by MSF mental health advisors and have been used in a range of MSF mental health programs for several years [12].

Quantitative change in functional status over a presentation was assessed for those receiving ≥2 counselling sessions by comparing self-rated functioning by the survivor at the initial and final counselling sessions. Presenting complaint status and change over time was recorded similarly to functional status for those receiving counselling.

The time to presentation was categorised as timely if it was within 72 hours after exposure to violence, as emergency oral contraception is most effective if taken as soon as possible after intercourse, and as 72 hours is the cut-off for effective provision of post-exposure HIV prophylaxis. Information was also collected on the source of referral (the source of information reported by the patient as instrumental in their awareness of and presentation to the service). Data were also collected on the provision of medical certificates which recorded the patient’s account of the incident and the health provider’s findings on medical examination. Age categories included separation at 16 years, the age of consent in PNG at the time of data collection.

### Statistical analyses

Population estimates for the programme’s catchment area for 2012 were based on the 2011 PNG census [13]. These figures, adjusted for the age distribution in urban areas (using the 2006 PNG Demographic and Health Survey [14]), were used to estimate the proportion of the population by age group and sex, presenting for care during the study period. The period prevalence’s for the relevant age groups (<5, 5–9 and 10–14) were adjusted for the duration of the study period (2.75 years) and the number of years covered by each age group (i.e. 5 years) in order to estimate the probability of a girl from this population presenting to the service prior to age 15.

All analyses, unless otherwise specified, were based on presentations; a single individual could have multiple presentations. χ^2^-squared or Fisher’s exact tests were used in initial descriptive analysis to assess the distribution of demographic, presentation and treatment characteristics by type of violence and age group, categorised as < 5 (prior to school age), 5 to 15 (as in PNG at the time of data collection the age of consent was 16 years), 16–24, 25–34, and > 34 years of age. Multiple variable adjusted analyses, also based on presentations, were carried out to assess factors associated with repeated presentation, and with timeliness of presentation for those presenting following sexual violence. Modified Poisson regression with robust variance estimation was used to estimate risk ratios (RR) in multiple variable analyses [15], accounting for within-patient correlation (i.e. multiple presentations by the same individual following different event(s) of violence).

Observations missing values for any outcome or explanatory variables were excluded. The results presented for each analysis include all of the variables considered *a priori* as potentially associated with the outcome of interest.

### Ethics

This paper presents an analysis of routinely collected program data, an essential component of ongoing evaluation and quality improvement of clinical services, including in MSF programs. In keeping with international standards [16], in order to facilitate such essential quality assurance processes while at the same time safeguarding patient confidentiality, the MSF ethical review board has determined requirements for exempting specific proposals addressing secondary analyses of routinely collected patient data from the requirement for formal human research ethical review [17, 18]. The MSF-OCA Research Committee assessed the current analysis as meeting these requirements. Specific written or verbal consent was therefore not sought from participants who contributed data to this analysis. Data were de-identified by the program data manager responsible for entering and analysing data for routine patient care and program purposes. This de-identified data was provided to the authors for the program evaluation reported in this paper. The same process and safeguards were applied to children who contributed data to the analysis. Ethical approval was also obtained from the Australian National University Human Research Ethics Committee. The data were used under approval for routinely collected data in operational research from the MSF-OCA Research Committee. The aggregate data are available in the paper. Because of ethical restrictions, individual-level data cannot be shared publicly. The data are available under the terms of MSF's data sharing policy, found at http://fieldresearch.msf.org/msf/handle/10144/306501.

## Results

### Demographic characteristics

Medical care was provided for 6,860 separate presentations by 5,892 individuals from August 2010 to April 2013. 28.8% of the presentations in this period were repeat presentations. That is, they were presentations by individuals who attended the service in the study period but had also presented at least twice in total over the period from January 2008 to April 2013.

Overall, an estimated 4.9% (95% CI:4.8%-5.0%) of females resident in the catchment area presented to the programme at least once from August 2010-April 2013. Females aged 25 to 29 years were most likely to present (10.2%, 95% CI:9.6–10.8%) (Table 1).Based on levels of presentation for those age groups between 0–14 years, we estimate that 7.0% of all female children in this population would present for care by 15 years of age, with the majority (77.9%) presenting following sexual violence.

**Table 1:** Proportion of female population presenting to programme at least once, by age group within the period August 2010-April 2013.

Age group	Patients (N)	Population distribution (%)	Estimated population size	August 2010-April 2013 period prevalence of presentation (95%CI*)
< 5	109	14.2	14,755	0.7 (0.6–0.9)
5–9	140	12.0	12,469	1.1 (1.0–1.3)
10–14	221	10.7	11,118	2.0 (1.7–2.3)
15–19	501	10.0	10,391	4.8 (4.4–5.3)
20–24	919	11.5	11,950	7.7 (7.2–8.2)
25–29	1062	10.0	10,391	10.2 (9.6–10.8)
30–34	860	8.3	8,625	10.0 (9.4–10.6)
≥ 35	1267	23.2	24,107	5.3 (5.0–5.5)
Total	5079	100	103,910	4.9 (4.8–5.0)

* 95% binomial exact confidence intervals for proportion of female population in age group presenting to programme.

The great majority of presentations (96.6%, N = 6,624) were female; however, male presentations more common in those <16 years of age compared to older age groups.

### Characteristics of violence

Across all 6,860 presentations, 61.5% (N = 4,219) reported IPV, 14.5% (N = 993) reported non-partner SV, 2.7% (N = 185) reported other forms of violence, and 21.3% (N = 1,463) reported past violence (Table 2). The violence reported by IPV patients included physical violence alone (71.9%), or physical violence associated with current sexual violence (9.5%) or with past sexual violence (18.7%).

**Table 2:** Characteristics of presentations, by type of violence, for presentations August 2010 to April 2013.

	N*	Intimate partner violence	Non-partner sexual violence	Other violence	Past violence	Total	χ^2^ / Fisher’s exact p-value**
		n = 4,219	n = 993	n = 185	n = 1,463	n = 6,860	
		%	%	%	%	%	
**Sex**							
Female	6,624	98.9	96.5	69.7	93.3	96.6	
Male	236	1.1	3.5	30.3	6.7	3.4	<0.001
**Age group**							
< 5	137	0.0	9.5	20.0	0.4	2.0	
5 to 15	566	0.2	40.1	43.2	5.4	8.3	
16 to 24	1,673	23.6	34.7	30.8	18.9	24.4	
25 to 34	2,670	46.2	10.3	3.8	42.2	39.0	
≥ 35	1,806	30.0	5.4	2.2	33.1	26.4	<0.001^
**Treatment facility**							
Angau Family Support Centre	6,242	90.1	98.3	84.9	90.5	91.2	
Urban health centres	599	9.9	1.7	15.1	9.5	8.8	<0.001
**Repeat presentations**							
Seen once	4,894	63.3	90.7	89.7	79.0	71.3	
Seen more than once	1,966	36.7	9.3	10.3	21.0	28.7	<0.001
**Reported perpetrator**							
Intimate partner	4,190	99.3	0.0	0.0	-	78.1	
Member of family / extended family	315	0.3	17.6	77.8	-	5.9	
Known person other than family	644	0.5	61.3	17.5	-	12.0	
Unknown person(s)	213	0.0	21.1	4.7	-	4.0	N/A
**Referred by**					-		
Self-reference / relative / friend / existing client	2,962	60.8	31.4	46.7	-	54.9	
Police	1,005	14.6	36.2	16.3	-	18.6	
Hospital / health facilities/ outreach workers	1,265	22.1	27.5	33.7	-	23.5	
Other	160	2.5	4.9	3.3	-	3.0	<0.001
**Time to presentation**							
≤ 72 hours	3,726	71.4	62.2	71.0	-	69.7	
>72 hours	1,622	28.6	37.9	29.0	-	30.3	<0.001
**Year**							
2010 (Aug-Dec)	1,017	14.6	13.6	11.9	16.8	14.8	
2011	2,171	31.7	35.7	33.0	28.5	31.7	
2012	2,953	42.5	39.6	43.2	47.0	43.1	
2013 (Jan-Aug)	719	11.2	11.2	11.9	7.7	10.5	0.115

*Numbers do not always sum to 6,860 due to missing data on the exposure variables.

**Comparing IPV and non-partner SV only.

^presentations in patients 16 years or older only.

There was significant variation in characteristics between IPV and non-partner SV presentation (Table 2). Male presentations were uncommon in both groups, but higher in non-partner SV. In those16 years and over presentations were more common in the older age groups for IPV than non-partner SV. Repeat presentation, reference by self or relative and presentation within 72 hours was also more common in IPV presentations. Presentations for IPV compared with non-partner SV did not vary significantly by year.

Almost half (49.5%) of those presentations reporting non-partner SV were children <16 years; 5.9% of those were male. Overall 86.9% of non-partner SV presentations were for rape, and 79.9% were perpetrated by someone known to the survivor, with an even higher proportion of known perpetrators in children under 16 years (87.8%). In a considerable proportion of non-partner SV, the sexual violence had occurred previously (15.1%) or was ongoing (6.2%), and such prior occurrence was significantly higher in children under 16 years (Table 3). Multiple perpetrators were reported in 22.8% of adult presentations for non-partner SV. Compared with presentations 16 years or older, more common amongst <16 year old presentations were male sex; sexual assault other than rape; and referral by health staff. Characteristics more common in those presentations 16 years old and over compared with younger age groups included repeat presentation to the service and reported perpetrator being unknown to the patient.

**Table 3:** Characteristics of non-partner sexual violence, by age group, for presentations August 2010 to April 2013.

	N*	< 5	5 to 15	16 to 24	25 to 34	≥35	Total	χ^2^/ Fisher’s exact p-value
		n = 94	n = 398	n = 345	n = 102	n = 54	n = 993	
		%	%	%	%	%	%	
**Sex**								
Female	958	94.7	94.0	98.3	100.0	100.0	96.5	
Male	35	5.3	6.0	1.7	0.0	0.0	3.5	0.002
**Repeat presentations**								
Seen once	901	98.9	94.0	89.9	80.4	77.8	90.7	
Seen more than once	92	1.1	6.0	10.1	19.6	22.2	9.3	<0.001
**Type of sexual violence**								
Rape	855	60.4	89.9	91.9	84.0	82.7	86.9	
Other	129	39.6	10.1	8.1	16.0	17.3	13.1	<0.001
**Frequency of sexual violence**								
Ongoing	58	6.7	9.6	3.6	4.3	0.0	6.2	
Repeated in past	141	21.3	19.4	12.0	5.4	10.0	15.1	
Single event	738	72.0	71.0	84.4	90.3	90.0	78.8	<0.001
**Perpetrator**								
Member of family / extended	173	23.2	27.2	12.0	2.9	5.7	17.8	
Known person other than family	595	64.6	62.6	60.1	53.9	66.0	61.2	
Unknown person(s)	205	12.2	10.2	28.0	43.1	28.3	21.1	<0.001
**Referred by**								
Self-reference / relative / friend / existing client	311	29.8	31.7	31.0	30.4	35.2	31.4	
Police/CIS	359	16.0	34.0	41.7	44.1	37.0	36.2	
Hospital / health facility / MSF workers / outreach	273	48.9	29.7	22.3	20.6	20.4	27.5	
Other	49	5.3	4.5	4.9	4.9	7.4	4.9	<0.001

*Numbers do not always sum to 993, due to missing values.

### Characteristics of medical care and counselling received

92.3% of IPV and 89.6% of non-partner SV presentations received medical treatment for physical injuries. The most common treatment for IPV was provision of analgesia for cuts, bruises and superficial injuries (56.5%), and 10.3% received suturing or orthopaedic care (Table 4). For non-partner SV, most common was provision of prophylaxes (58.4%). Of these presentations, 49.9% were offered HIV post-exposure prophylaxis. Of the 9.5% of IPV patients reporting IP-associated sexual violence, 44.2% were offered HIV prophylaxis. In both cases, the main reason for not offering HIV prophylaxis was lack of timely presentation.

**Table 4:** Characteristics of medical care (excluding counselling care) provided, by type of violence (excludes past violence), for presentations August 2010 to April 2013.

	Intimate partner violence	Non-partner sexual violence partner	Other violence	Total	Chi2 p-value
	% (N)	% (N)	% (N)	% (N)	
No medical treatment*	7.7 (324)	10.4 (103)	14.0 (25)	8.4 (452)	
Wound care	18.6 (783)	4.5 (45)	16.8 (30)	16.0 (858)	
Suturing	5.3 (223)	1.1 (11)	2.8 (5)	4.4 (239)	
Orthopaedic care	5.0 (210)	0.0 (0)	3.9 (7)	4.0 (217)	
Pain medication	56.5 (2,378)	18.7 (186)	40.8 (73)	49.0 (2,637)	
Prophylaxis	4.1 (172)	58.4 (580)	6.2 (11)	14.2 (763)	
Other**	2.7 (117)	6.8 (68)	15.7 (28)	3.9 (213)	
Total	100 (4,207)	100 (993)	100 (179)	100 (5,379)	<0.001

* Patient did not receive medical treatment for physical injuries.

**Other includes: observation, treatment of infectious disease or antibiotics.

73.7% (N = 4,343) of patients presenting to the service received counselling, with a significantly lower proportion of children counselled (31.6% of children under 5years and 59.2% of 5–10 year olds) (Chi2 p-value <0.000). The number of counselling sessions received varied significantly by type of precipitating event, with presentations for non-partner SV more likely to receive multiple counselling sessions (59%) compared to presentations for IPV (37%) or other violence (26.4%) (Chi2 p-value <0.001) (Table 5). Of those patients over 16 years of age who received more than one counselling session, the majority reported improved functioning (81.2%) and improvement in the presenting complaint (85.5%).There was significant variation in improvement in presenting complaint by type of violence, with the highest improvement reported in those presenting for non-partner sexual violence (92.1%, chi2 p-value 0.001). Functional improvement did not vary significantly by type of violence (Table 5).

**Table 5:** Number of counselling sessions and change in functional capacity and severity of presenting complaint, by type of precipitating event, for presentations by patients over 16 years receiving counselling August 2010 to April 2013.

	N*	Intimate partner violence	Non-partner sexual violence	Other violence	Total	Chi2 /Fisher’s exact p-value
		N = 3,848	N = 466	N = 95	N = 4,409	
		%	%	%	%	
**Number of counselling sessions**						
1	2,641	62.0	39.7	73.7	59.9	
2–3	1,503	32.1	47.0	23.2	34.1	
>3	265	5.2	13.3	3.2	6.0	<0.001
Total						
**Type of exit**						
Left prior to completing treatment	2,697	62.0	54.1	57.9	61.1	
Discharged	1,719	38.0	45.9	42.1	38.9	0.003
Change in functioning*						
Improved	1,434	80.4	85.0	84.0	81.2	
Unchanged	219	12.9	10.0	12.0	12.4	
Worse	114	6.8	5.0	4.0	6.5	0.466
Change in presenting* complaint						
Improved	1,510	84.1	92.1	88	85.5	
Unchanged	176	11.1	3.9	12.0	10.0	
Worse	81	4.8	3.9	0.0	4.6	0.001

* For those aged receiving more than one counselling session (N = 1,752).

### Patterns of services use by catchment population

**Fig 1:**
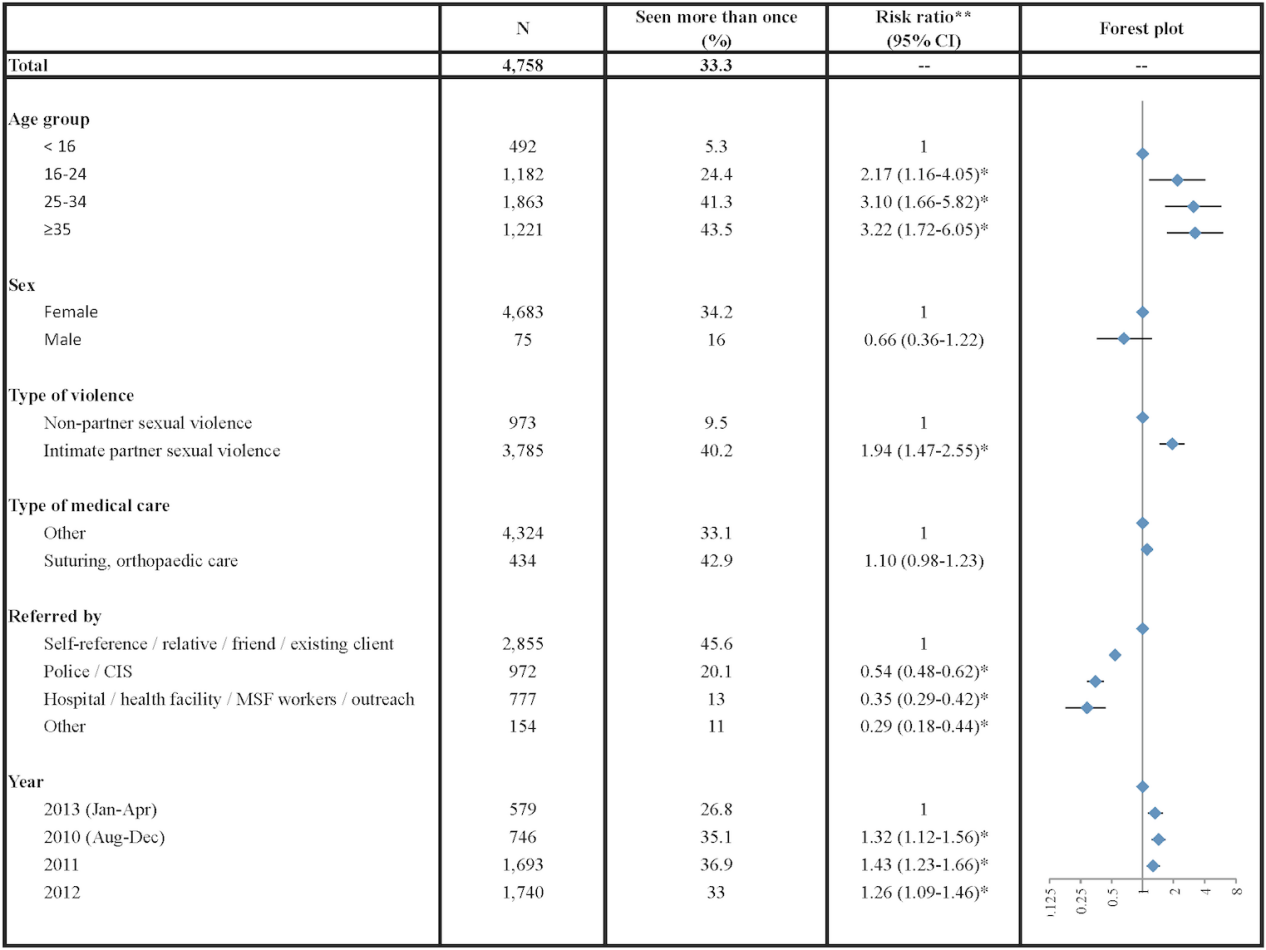
Factors associated with presenting more than once vs once, for presentations associated with intimate partner violence or non-partner sexual violence.^†^ ^†^ Patients presenting more than once since Jan 2008, for those presentations from Aug 2010 to April 2013 to the FSC. *Multiple variable analyses of presentations to the Family Support Centre adjusted for age, sex, type of violence, type of medical care, source of referral, and year. Patients presenting for past violence were not included as data were not included on referral source. These analyses were conducted on the sample of 4,903 presentations with complete data on these variables. **Horizontal bars indicate 95% confidence intervals.

In adjusted analysis, older age groups were 2–3 times more likely to present to the FSC repeatedly compared to those less than 16 years (Fig 1). Patients were also more likely to present multiple times if they experienced IPV compared to non-partner SV (RR:1.94, 95% CI:1.47–2.55), and if they were referred by themselves or a friend, compared to by the police (RR:0.54, 95% CI:0.48–0.62), hospital (RR:0.35, 95%CI:0.29–0.42), or other service (RR:0.29, 95% CI:0.18–0.44).

**Fig 2:**
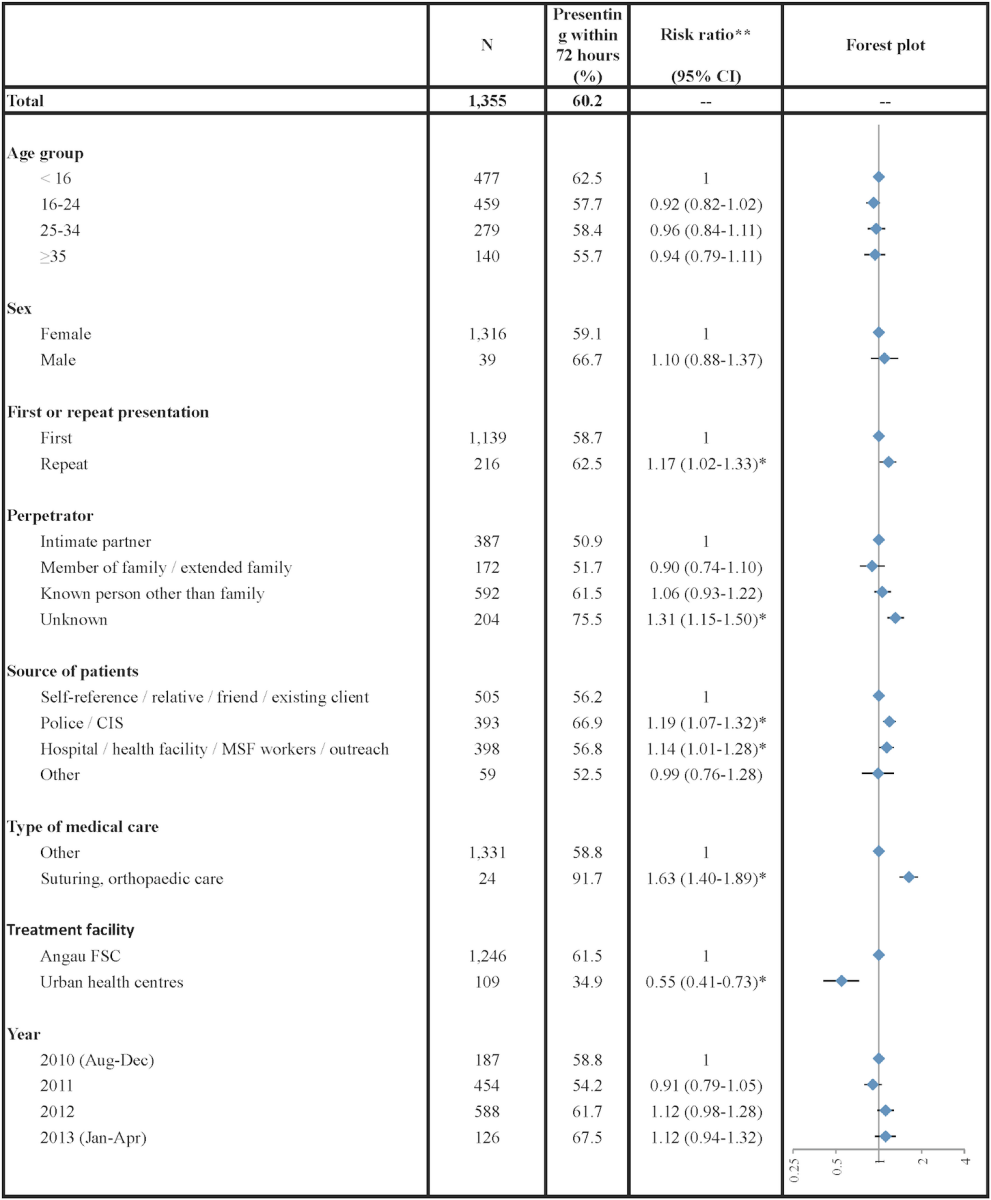
Factors associated with presentation <72 hours vs ≥72 hours after sexual violence. * Multiple variable analyses of timely for presentations following sexual violence (both non-partner and intimate partner violence) adjusted for age, sex, first or repeat presentation, perpetrator, place of residence, source of referral, treatment facility, and year. These analyses were conducted on the sample of 1,355 presentations with complete data on these variables. **Horizontal bars indicate 95% confidence intervals.

Overall, 60.2% of patients presenting following current sexual violence (IPV with current SV or non-partner SV) did so within 72 hours of this violence occurring (Fig 2). In adjusted analysis, timely presentation was more likely if it was a repeat presentation (RR:1.17, 95% CI:1.02–1.33); if the violence was perpetrated by an unknown person, compared to an intimate partner (RR:1.31, 95% CI:1.15–1.50); if referred by the police (RR:1.19, 95% CI:1.07–1.32) or health care workers (RR:1.14, 95% CI:1.01–1.28), rather than self-referral; or if suturing or orthopaedic care was required, compared to more minor medical interventions (RR:1.63, 95% CI:1.40–1.89). Timely presentation was about half as likely among those presenting to urban health centres, compared to the Angau FSC (RR:0.55, 95% CI:0.41–0.73).

### Referral to the FSC

Overall, the most common source of new patients was self-referrals or referrals made through friends and/or relatives (42.4%). 24.2% were referred from other health services, 16.5% by police, but very few were referred from legal services, welfare or child protection departments, or non-governmental organisations. Overall, health staff completed a medical certificate for 55.3% of presentations. This varied by the type of violence, with 91.6% of presentations for non-partner SV having a certificate completed compared with 34.5% of IPV and 25.6% of presentations associated with other types of violence.

## Discussion

In most countries including PNG, IPV is the most prevalent form of gender-based violence, consistent with our findings [19, 20]. In a 2008 study of women presenting to antenatal care in PNG, 44% reported ever experiencing IPV, and 70% of those had never accessed support services [21]. Our analysis demonstrates that if free, high-quality, accessible services are available, survivors of IPV will utilise care. We cannot estimate directly from our data the proportion of women experiencing intimate partner violence in the study community who would present to the health service over a lifetime. However, the estimate of 4.9% of women presenting over a 2.8 year period suggests a high lifetime presentation rate. All those who presented had access to a complete package of care for sexual and gender-based violence, at no cost, and without the need for referral. The few other services for survivors in PNG, where available, often charge fees and require referral documentation, suggesting that the low levels of service uptake previously reported may be, at least in part, due to lack of services, particularly those that are free and easy to access.

Many survivors of non-partner sexual violence also presented for care. In a study of women attending antenatal and HIV voluntary testing and counselling services across four provinces of PNG, 9.7% of the sample (n = 114) reported rape prior to the age of 16 [4]. Our study estimates a presentation rate of 7.0% of females aged 0–14 in the catchment population during the analysis period, almost all of whom presented for rape, suggesting that where services are available for child sexual abuse survivors, a considerable proportion will present, in particular those experiencing severe forms of abuse such as rape. Community outreach activities are likely to have contributed to the high proportion of self and family referrals.

The characteristics of non-partner SV reported in this analysis are disturbing. Almost all presentations were for rape, and in many cases, the violence had occurred previously or was ongoing, and involved multiple perpetrators and the use of death threats. All such factors increase not only the physical but the psychological trauma inflicted by such violence. The need for appropriate mental health services to address such trauma is great [7]. The value of counselling is suggested by improved self-reported functioning and psychosocial status in patients receiving multiple sessions. Access to specialist mental health counselling services are very limited or non-existent in most parts of PNG. Services, when available, are provided by general health workers and focus on mood, substance and psychiatric disorders [22]. There is an urgent need to addresses these gaps in the provision of high-quality, confidential mental health counselling services through training of specialist mental health staff and ensuring allocated positions for such staff within government bodies. A specific group that needs even greater focus is child survivors of sexual violence, who constituted almost half of presentations following non-partner SV, but a lower proportion of those receiving counselling.

Presentation for sexual violence may be delayed due to lack of awareness of availability or need for care [23]. Our study supports this conclusion. Presentation was timelier in those who were already aware of the service through previous presentations or through other service providers. It was also timelier for those who presented to the specific gender-based violence services of the FSC rather than those identified through screening of general presentations to urban health centres. This suggests areas where educational messages could add value, such as community-based campaigns on the availability and value of early presentation to access effective services for preventing unwanted pregnancies and HIV infection.

Those presenting for IPV were more likely to present repeatedly and were more likely to present with major injuries requiring suturing or orthopaedic care than those presenting for non-partner SV. The proportion of IPV clients reporting sexual violence and non-partner SV offered HIV prophylaxis were similar. The low rate of referral from police among IPV, compared to non-partner SV, survivors suggests that policing services are less accessed by IPV patients. Medical certificates are considered by service providers as important in supporting criminal proceedings or compensatory claims in village courts [24]. Our finding that medical certificates were more likely to be provided in cases of non-partner SV compared with IPV again suggests that there is a considerable bias towards pursuing such avenues in cases of non-partner SV compared with IPV. Those presenting for non-partner SV were significantly more likely to remain in counselling for multiple sessions, report improvements in presenting complaint, and be discharged rather than default than those presenting for IPV. IPV patients are at ongoing risk from their partner if they continue to remain in the relationship, whereas a lower proportion of adult patients presenting following non-partner SV are exposed to the perpetrator and therefore at risk on an ongoing basis. The ongoing exposure of IPV patients to violence is likely to underlie the lower retention and effectiveness of counselling in this group, as well as the higher levels of repeat presentation and more severe forms of physical injury.

Safety assessment or planning interventions were limited to referral of patients to appropriate services such as community welfare, policing or legal services. Although data on referrals were not collected over the period analysed, previous data from the programme shows that the proportion of patients referred from the FSC to other services dropped significantly from January 2008 to April 2010 (from 50% to 15% for police, 12% to 0.2% for legal services and 8.3% to 0.1% for welfare services), suggesting that such referrals came to be seen over time as of limited value. This is concerning in a context where much of the violence is perpetrated by known individuals and therefore is likely to recur unless resolved definitively. As studies from other settings have shown, medical and counselling services for survivors must be linked to effective services in other sectors such as policing and the judicial system if they are to improve long-term survivor outcomes [25].

This study presents findings from patients presenting for care to a sexual and gender-based violence programme that targeted intimate partner and/or sexual violence, so our results reflect this selective targeting. An additional limitation is that catchment population data was from 2011 census, and therefore would not reflect more recent changes that may have occurred. This, along with the use of health facility based data, limits the generalizability of our findings to population-level inferences on prevalence of different forms of violence. However, as discussed above, our findings are consistent with available prevalence data from PNG.

## Conclusions

Our analyses demonstrate that, in settings such as PNG with a known high prevalence of gender-based violence, when free, confidential, accessible medical and counselling services are provided, many survivors will present for care. Those who do present receive best-practice medical care and counselling, in particular if they present in a timely way and are retained in care. The majority who received two or more sessions of counselling had improvement in self-reported functioning, with the most improvement seen in those reporting non-partner SV. However, high levels of IPV and child sexual violence by known perpetrators suggests that effective policing, protection and legal services will also be required alongside health services if survivors are to escape the cycle of violence.
